# Neuropathic Low Back Pain and Burnout among Hungarian Workers

**DOI:** 10.3390/ijerph18052693

**Published:** 2021-03-07

**Authors:** Kornél Mák, Krisztián Kapus, Gábor Tóth, Dávid Hesszenberger, Marietta Pohl, Gabriella Pusch, Éva Fejes, Gergely Fehér, Antal Tibold

**Affiliations:** 1 Centre for Occupational Medicine, Medical School, University of Pecs, 7624 Pecs, Hungary; mak.kornel@bacskiskun.hu (K.M.); kapusk@gmail.com (K.K.); dtg1019@gmail.com (G.T.); pohlmarietta@bmvk.hu (M.P.); efchengirl@gmail.com (É.F.); tibold.antal@pte.hu (A.T.); 2 Bajai Szent Rókus Hospital, 6500 Baja, Hungary; 3 Department of Laboratory Medicine, Medical School, University of Pecs, 7624 Pecs, Hungary; david.hesszenberger@aok.pte.hu; 4 Department of Neurology, Medical School, University of Pecs, 7624 Pecs, Hungary; puschgabriella74@gmail.com; 5 Hospital of Komlo, 7300 Komlo, Hungary; 6 Neurology Outpatient Clinic, EÜ-MED KFT, 7300 Komlo, Hungary

**Keywords:** low back pain, neuropathic pain, burnout, prevalence, risk factor, multivariate analysis

## Abstract

Burnout is an increasingly prevalent syndrome mainly involving those working in human services. Although it is categorized as an occupational phenomenon and not as a medical condition, it seems to be strongly associated with several diseases such as pain syndromes. However, no studies examined the association between neuropathic low back pain and burnout. This questionnaire-based study was carried out between April 2019 and March 2020 in three main sites among teachers, social workers and healthcare workers. Demographic criteria included age, gender, marital status, number of children, type of work, years spent with work, work schedule, legal relation, secondary employment. Included diseases were diabetes, hypertension, ischemic heart disease, generalized pain (pain involving more than one area of the body) and depression. Low back pain was assessed by the painDETECT questionnaire, burnout was measured with the Maslach Burnout Inventory (MBI) and depression was measured by the Beck Depression Inventory. Dysfunctional attitudes were also recorded. Overall, 1500 questionnaires were successfully delivered and 1141 responses received (response rate of 76%). Three hundred social workers, 399 teachers, 339 paramedics, 35 doctors and 68 medical attendants have completed our survey. In a multivariate analysis including of all factors (demographic criteria, burnout, depression, dysfunctional attitudes, comorbidity etc.) neuropathic low back pain was associated with age > 62 (OR = 3.981, *p* = 0.01), number of children ≥ 2 (OR = 2.638, *p* = 0.003), job type (being a social worker) (OR = 6.654, *p* < 0.001), burnout (OR = 2.577, *p* < 0.001), current depression (OR = 2.397, *p* < 0.001), and suffering from generalized pain (OR= 4.076, *p* < 0.001). This is the first study showing the association of burnout and neuropathic low back pain, which is the most common cause of disability. Based on our results neuropathic low back pain and burnout have similar risk factors and consequences which raises the possibility of similar pathophysiology.

## 1. Introduction

Burnout is an increasingly prevalent syndrome resulting from situational, personal and professional stress (mainly driven by workplace stress) and can be characterized by emotional exhaustion, depersonalization, and reduced personal accomplishment [1]. The most vulnerable people are those working in human services but it is broadened to many other occupations. It has great impacts on the individual and on the society as it may lead to undesirable consequences such as emotional depletion, loss of energy, dehumanization, detachment from work, feeling of inadequacy, reduced productivity and coping skills thereby involving workers, their colleagues, clients, and families, the work environment and the organizations [2].

The most important risk factors of burnout are gender, marital status, work environment, interpersonal and professional conflicts, emotional distress, and low social support [3].

Although it is categorized as an occupational phenomenon and not as a medical condition, it seems to be strongly associated with diabetes, cardiovascular disorders, chronic musculoskeletal pain, headaches, changes in pain experiences, gastrointestinal issues, respiratory problems, severe injuries and mortality below the age of 45 years as well as with psychiatric complications such as insomnia, depressive symptoms and mental disorders based on a recent systematic review [2]. The physical consequences were mostly driven by cardiovascular disorders and pain syndromes [2].

Chronic pain is a devastating disease, one of the leading causes of disability, which can affect 10–20% of the population [4,5]. Neuropathic pain, which originates from injury or malfunction to the peripheral or central nervous system resulting in maladaptive changes in neurons along the nociceptive pathway can be detected about 30% of chronic pain patients [5].

Lower back and neck pain are the leading causes of disability in the jobholder population based on recent data [4]. Lower back pain is a complex, heterogeneous condition, where both nociceptive and neuropathic pain mechanisms may be involved, and the rate of neuropathic component can as high as 55% [6].

Similar to burnout, emotional aspects also play an important role in the development of neuropathic pain [7]. Impaired familial and social interactions have been shown to be associated with both burnout and neuropathic pain [3,7,8].

Despite of the possible association of chronic pain syndromes and burnout, based on our knowledge and literature search, no study focused on the association between neuropathic low back pain and burnout.

The aim of our prospective cross-sectional study was (a.) to detect the prevalence and risk factors of neuropathic pain, (b.) to examine the role of risk factors of burnout on neuropathic pain and (c.) to assess the connection between burnout and neuropathic pain among human service workers including educational, healthcare or social area.

## 2. Patients and Methods

This questionnaire based cross-sectional study was carried out between April 2019 and March 2020, patients were recruited from three cities (cities of Komlo, Pecs and Kecskemet). The study was approved by the Ethical Committe of the University of Pecs (reference number: PTE/96773-2/2018).

Paper based questionnaires were posted to teachers in the educational region of Kecskemét, Pécs and Komló (77 schools), to the social workers of Ágota Foundation (1 major site in Kecskemét and 37 minor sites in Central Hungary) and to the healthcare workers employed in the hospitals of Komló, Kecskemét and Pécs (3 sites).

Inclusion criteria were working with human services as a healthcare worker, social worker or teacher, being between 18 and 65 years of age and being employed at the time of the study apart from the type of employment (public servant, subcontractor etc.).

Exclusion criteria were being under 18 or over 65 years of age, having disability, having severe uncontrolled disease, being on permanent leave or refusing to participate in the study.

Demographic criteria included age, gender, marital status, number of children, type of work, years spent with work, work schedule, legal relation, secondary employment.

Included diseases were diabetes, hypertension, ischemic heart disease, generalized pain (pain involving more than one area of the body) and depression.

Low back pain was assessed by the painDETECT questionnaire, which is a simple, patient-based, easy-to-use screening questionnaire developed by Freynhagen and his co-workers in cooperation with the German Research Network on Neuropathic Pain. It can determine the prevalence of neuropathic pain components both in individual low back pain patients and in heterogeneous cohorts of such patients [9,10]. We used the following cut-off values as suggested by the authors: 0–12 neuropathic pain is unlikely, 13–18 probable neuropathic pain, 19–38 neuropathic pain is very likely.

Burnout was measured with the Maslach Burnout Inventory (MBI), which is an introspective psychological inventory consisting of 22 items pertaining to three dimensions of burnout: emotional exhaustion, depersonalization, and personal accomplishment [11]. Responses are marked on a seven-point Likert scale (0 meaning “never” and 6 meaning “every day”) and then summed. The first nine items assess emotional exhaustion (EE), the second five item assess depersonalization (DP), the seven last items assess reduced personal accomplishment (PA). We defined EE as EE score ≥ 27, DP as DP score ≥ 10, and PA as PA score ≤ 33. The overall burnout was defined as EE score ≥ 27 and/or DP score ≥ 10 [12]. For EE and DP, subscale score of 0–9 was categorized as “no to low burnout” and subscale score of 10–18 was regarded as “moderate burnout.” It was the opposite for PA because higher PA scores indicate lesser burnout [12]. This copyright protected questionnaire can be downloaded in Hungarian translation from the website [13].

Depression was measured by the Beck Depression Inventory which is a 21-question multiple-choice self-report inventory, one of the most widely used psychometric tests for measuring the severity of depression. It is composed of relating symptoms-based items such as hopelessness, irritability, guilt or feelings of being punished, fatigue, weight loss, and lack of interest in sex [14,15]. Each item is rated from 1 to 4. We used the following cut-off values: 0–9 normal, 10–18: mild, 19–27: moderate, 28–36: severe depression.

The Dysfunctional Attitude Scale (DAS) is a 40-item instrument that is designed to identify and measure cognitive distortions, particularly distortions that may relate to or cause depression [16,17]. Each item is rated from 1–4. The total score is the sum of the 40-items with a range of 40–280. The higher the score, the more dysfunctional attitudes an individual possesses

Statistical analysis: Data were evaluated as means ± SD (standard deviation) the chi-square test, distribution ratios and the Pearson’s Rank-Order Correlation. Data were analyzed using descriptive statistics, linear regression, correlation, multivariate analysis of variance, and factor analysis. Analysis of variance (ANOVA) was used in neuropathic low back pain analysis. Logistic regression analysis was used to determine the significance of the included parameters as independent risk factors in the development of neuropathic low back pain. For all odds ratios, an exact confidence interval (CI) of 95% was constructed in our study. Logistic regression analyses were performed using the statistical package of SPSS 11.0 (SPSS, Chicago, IL, USA).

## 3. Results

Overall, 1500 questionnaires were successfully delivered and 1141 responses received (response rate of 76%). Three hundred social workers, 399 teachers, 339 paramedics, 35 doctors and 68 medical attendants have completed our survey.

643 males (56.4%) and 498 females (43.6%) participated in our study. About one third of the examined workers was between 36–45 years of age (31.5%). 764 people (67%) were married or lived in a relationship. The number of childless workers was 65 (5.7%). 150 participants (13.1%) had secondary employment. The vast majority (44.2%) of our study population has been working for 11–30 years. Baseline data can be seen in Table 1.

Two hundred seventy-eight (24.4%) participants had hypertension, and sixty-six (5.8%) were diabetic. Ischemic heart disease can be detected in 161 (14.1%) workers. Two hundred twenty (19.3%) participants suffered from generalized pain (pain involving more than one area of the body). Thirty-nine (3.4%) persons had a history of depression (Table 2).

Low back pain occurred in 630 workers (55.2%): 264 (23.1%) had neuropathic low back pain (final score above 19 points), 366 workers (32.1%) had probable neuropathic pain (final score 13–18 points) and 511 (44.8%) workers had nociceptive low back pain (final score: 0–12 points) (Table 3).

The prevalence of significant burnout among the study population was 81.4%. The mean burnout score was 57.6 (SD = 16.2); 212 workers had mild (18.6%), 884 had moderate (77.5%) and 45 had severe (3.9%) burnout (Table 2).

ANOVA analysis showed that neuropathic low back pain was associated with age over 62 (*p* = 0.035), having children ≥ 2 (*p* = 0.005), being a social worker (*p* = 0.03), and having a secondary employment (*p* = 0.014) (Table 3).

Neuropathic low back pain was also associated with hypertension (*p* = 0.006), diabetes (%, *p* < 0.001), history of depression (*p* = 0.014) and generalized pain (*p* = 0.044) based on ANOVA analysis (Table 4).

Moderate and severe depression as well as dysfunctional attitudes were also associated with neuropathic low back pain (*p* < 0.05 in all cases) (Table 4). The presence of depression and dysfunctional attitudes were also significantly associated with neuropathic low back pain (R = 0.38, *p* = 0.004 and R = 0.327, *p* < 0.001, respectively).

Severe burnout was also significantly associated with neuropathic low back pain (*p* = 0.012) (Table 4). There was a weak but significant association between the presence of any burnout and neuropathic low back pain (R = 0.169, *p* < 0.001).

In a multivariate analysis including of all factors (demographic criteria, burnout, depression, dysfunctional attitudes, comorbidity, etc.) neuropathic low back pain was associated with age > 62 (OR = 3.981, *p* = 0.01), number of children ≥ 2 (OR = 2.638, *p* = 0.003), job type (being social worker) (OR = 6.654, *p* < 0.001), burnout (OR = 2.577, *p* < 0.001), current depression (OR = 2.397, *p* < 0.001), and suffering from generalized pain (OR = 4.076, *p*< 0.001).

## 4. Discussion

This is the first study showing the association of burnout and neuropathic low back pain in a large cohort of workers.

Burnout is an increasingly prevalent syndrome mainly involving those working with human services; therefore, we targeted workers from the health, social and educational sectors [18].

Despite of intensive research on the topic, burnout is still labeled as an occupational disease and not as a medical condition; however, it seems to be associated with physical (such as cardiovascular disorders, diabetes, respiratory symptoms, sudden death etc.), psychological (depression, anxiety, substance abuse etc.), and occupational consequences (such as chronic pain syndromes which are amongst the leading causes of disability) [2,19,20].

The rate of low back pain was more than 50% in our population which is slightly above the estimated rates comparing to a previous study from Hungary [21]. One in five low back pain patients suffered from neuropathic pain and the rate of mixed pain (containing both nociceptive and neuropathic components) was similar. This is in concordance with previous findings, the rate of neuropathic low back pain varied from 16% to 55% [5].

In our survey neuropathic pain was associated with age, there was a 3.98-fold increase over 62, and living in a larger family (having children ≥ 2) also has a 2.6-fold increase in developing neuropathic low back pain. Job type (being a social worker) and secondary employment were also strongly associated with this condition in a univariate analysis; being a social worker has a six-fold increase of risk alone. In a Canadian study neuropathic pain was associated with low income, unemployment, smoking, and being unmarried, which are slightly similar to our findings [22]. Social workers usually have lower income compared to other participants of the study, and having secondary employment also reflects being involved circumstances. Interestingly, age, gender, marital status, and difficult working conditions also were independent risk factors of burnout based on a recent meta-analysis [23].

We also found significant association among neuropathic low back pain, depression, diabetes and hypertension. Hypertension and cardiovascular diseases have been previously documented as being associated with neuropathic pain [22,24,25]. Diabetes is the most important risk factor of developing neuropathic pain, and this type of pain is also strongly associated with mood disorders [2,26]. However, a multivariate analysis could not confirm their role as significant contributors.

Dysfunctional attitudes are usually associated with depression; however, they are strongly related to burnout [27]. Psychological, psychobehavioral and psychosocial factors also play relevant role in pain perception; therefore, dysfunctional attitudes, depression, burnout and neuropathic pain may share a common root as there is a strong association among them in our study [28].

We also found a four-fold increase in the risk of developing neuropathic low back pain among those suffering from generalized pain, which underlines the involvement of pain perception pathways.

Based on our results, neuropathic low back pain shared similar risk factors and consequences with burnout. Burnout was also a significant risk factor of neuropathic low back pain, workers suffering from burnout has an approximately 2.5-fold risk to suffer from neuropathic low back pain based on our results. The association can be bidirectional because burnout is mainly due to chronic occupational stress and work overload can result in the activation of the autonomic nervous system (ANS) and the hypothalamic-pituitary-adrenal (HPA) axis (which results in overactivation of vital functions such as blood pressure or heart rate), and this can be associated with low-grade inflammation, blood coagulation changes, depression of the immune system, sleep disorders, and adoption of poor health behaviors, such as overweight, smoking, excessive alcohol consumption and lack of physical activity [2,22,27,28,29,30]. This sympathetic hyperactivity and biochemical changes may play a role in the development of neuropathic pain also [31].

Secondly, neuropathic pain, which is caused by a lesion or malfunction of the somatosensory system is associated with imbalances between excitatory and inhibitory somatosensory signaling, alterations in ion channels and variability of pain processing pathways [32]. Imaging studies have confirmed altered activity of afferent and descending pain pathways, as well as atrophy of different pain perception regions of the brain, which can result in sympathetic overload leading to cardiovascular diseases and psychiatric symptoms [4,33]. It is supported by a Danish multicenter study, in which neuropathic pain was associated with depression and unfavorable cardiovascular outcome [34].

## 5. Conclusions

In summary, this is the first study showing the association of burnout and neuropathic low back pain, which is the most common cause of disability. Based on our results, neuropathic low back pain and burnout share similar risk factors and consequences, which raises the possibility of similar pathophysiology. We do not have a proper explanation to this phenomenon, but the activation of sympaticoadrenal system leading to katecholamine overload can probably be responsible for this association.

Finally, our article has some limitations. Although it was a prospective cross-sectional study in nature including more than 1000 workers, it was not representative of burnout and neuropathic pain neither in the general nor in the human services population. As it was a paper-based survey, physical examination was not carried out (such as screening for neuropathic pain in pending cases for example with DN4). We had no detailed medical information about the previous diseases and medication of the included workers as well as about the duration of low back pain and its treatment modalities. History of neuropathic low back pain was also not recorded. The above-mentioned limitations may influence our findings. Finally, follow-ups were not carried out.

## Figures and Tables

**Table 1:** Baseline characteristics of the study population.

(N = 1141)	%
**Gender**	
Female	43.6 (498/1141)
Male	56.4 (643/1141)
**Age**	
18–25 years	8.0 (91/1141)
26–35 years	15.0 (171/1141)
36–45 years	31.5 (359/1141)
46–55 years	28.9 (330/1141)
56–62 years	12.9 (148/1141)
more than 62 years	3.7 (42/1141)
**Marital Status**	
single	20.1 (230/1141)
relationship	19.2 (219/1141)
married	47.8 (545/1141)
divorced/widow	12.9 (147/1141)
**Number of Children**	
have no child	5.7 (65/1141)
1 child	32.6 (372/1141)
2 children	28.5 (325/1141)
more than 3 children	33.2 (379/1141)
**Type of Work**	
medical clerk	2.2 (25/1141)
assistant	10.4 (119/1141)
nurse	11.8 (135/1141)
doctor	3.1 (35/1141)
other health care worker	5.2 (60/1141)
swabber	2.7 (30/1141)
economical-technical workers	3.3 (38/1141)
social worker	26.3 (300/1141)
teacher	35.0 (399/1141)
**Years Spent with Work**	
1–12 months	7.1 (81/1141)
1–5 years	14.8 (169/1141)
6–10 years	14.5 (165/1141)
11–20 years	22.2 (253/1141)
21–30 years	22.0 (251/1141)
31–40 years	16.6 (190/1141)
more than 40 years	2.8 (32/1141)

**Table 2:** Comorbidity, dysfunctional attitudes, depression and burnout in the study population.

(N = 1141)	%
**Comorbidity**	
diabetes	5.8 (66/1141)
hypertension	24.4 (278/1141)
ischemic heart disease	14.1 (161/1141)
generalized pain	19.3 (220/1141)
history depression	3.4 (39/1141)
**Dysfunctional Attitudes**	
normal	28.9 (330/1141)
mild	57.7 (658/1141)
moderate	11.8 (135/1141)
severe	1.6 (18/1141)
**Current Depression**	
normal	39.8 (454/1141)
mild	48.3 (551/1141)
moderate	9.2 (105/1141)
severe	2.7 (31/1141)
**Burnout**	
mild	18.6 (212/1141)
moderate	77.5 (884/1141)
severe	3.9 (45/1141)

**Table 3:** Demographic factors associated with neuropathic low back pain, * *p* < 0.05.

	Nociceptive Pain N = 511	Unclear Pain N = 366	Neuropathic Pain N = 264
**Gender**			
Female	43.2 (221/511)	28.9 (106/366)	64.8 (171/264)
Male	56.8 (290/511)	71.1 (260/366)	35.2 (93/264)
**Age**			
18–25 years	7.8 (40/511)	7.7 (28/366)	8.7 (23/264)
26–35 years	15.6 (80/511)	15.3 (56/366)	13.3 (35/264)
36–45 years	30.9 (158/511)	30.4 (111/366)	34.1 (90/264)
46–55 years	28.0 (143/511)	31.7 (116/366)	26.5 (70/264)
56–62 years	14.1 (72/511)	11.8 (43/366)	12.5 (33/264)
more than 62 years	3.6 (18/511)	3.1 (11/366)	4.9 (13/264) *
**Marital Status**			
single	11.1 (115/511)	18.0 (66/366)	18.6 (49/264)
relationship	18.0 (92/511)	20.5 (75/366)	19.7 (52/264)
married	46.8 (239/511)	49.7 (182/366)	46.9 (124/264)
divorced/widow	24.1 (65/511)	11.8 (43/366)	14.8 (39/264)
**Number of Children**			
have no child	11.2 (57/511)	1.9 (7/366)	0.4 (1/264)
1 child	34.1 (174/511)	35.0 (128/366)	26.8 (71/264)
2 children	27.9 (143/511)	26.0 (95/366)	33.0 (87/264) *
more than 3 children	26.8 (137/511)	37.1 (136/366)	39.8 (105/264) *
**Type of Work**			
medical clerk	3.5 (18/511)	0.8 (3/366)	1.5 (4/264)
assistant	17.6 (90/511)	3.8 (14/366)	5.7 (15/264)
nurse	13.1 (67/511)	10.9 (40/366)	10.6 (28/264)
doctor	6.1 (31/511)	1.1 (4/366)	0.0 (0/264)
other health care worker	9.6 (49/511)	1.9 (7/366)	1.5 (4/264)
swabber	3.3 (17/511)	1.6 (6/366)	2.7 (7/264)
Economical-technical workers	5.1 (26/511)	2.5 (9/366)	1.2 (3/264)
social worker	12.5 (64/511)	29.2 (107/366)	48.8 (129/264) *
pedagogue	29.2 (149/511)	48.2 (176/366)	28.0 (74/264)
**Years Spent with Work**			
1–12 months	9.0 (46/511)	5.7 (21/366)	5.3 (14/264)
1–5 years	15.9 (81/511)	13.9 (51/366)	14.0 (37/264)
6–10 years	15.7 (80/511)	13.4 (49/366)	13.6 (36/264)
11–20 years	18.2 (93/511)	26.8 (98/366)	23.5 (62/264)
21–30 years	22.5 (115/511)	20.2 (74/366)	23.5 (62/264)
31–40 years	16.2 (83/511)	16.7 3(61/366)	17.4 (46/264)
more than 40 years	2.5 (13/511)	3.3 (12/366)	2.7 (7/264)
**Secondary Employment**			
No	87.3 (446/511)	88.8 (325/366)	83.3 (220/264)
Yes	12.7 (65/511)	11.2 (41/366)	16.7 (44/264) *

**Table 4:** Somatic and mental factors associated with neuropathic low back pain, * *p* < 0.05.

	Nociceptive Pain N = 511	Unclear Pain N = 366	Neuropathic Pain N = 264
**Depression**			
normal	40.5 (207/511)	42.3 (155/366)	34.8 (92/264)
mild	50.7 (259/511)	49.7 (182/366)	41.7 (110/264)
moderate	6.8 (35/511)	6.0 (22/366)	18.2 (48/264) *
severe	1.8 (10/1511)	2.0 (7/366)	5.3 (14/264) *
**Dysfunctional Attitudes**			
normal	45.6 (235/511)	20.5 (75/366)	7.6 (20/264)
mild	45.4 (232/511)	69.1 (253/366)	65.5 (173/264)
moderate	8.0 (41/511)	9.0 (33/366)	23.1 (61/264) *
severe	1.0 (36/511)	0.5 (5/366)	3.8 (10/264) *
**Burnout**			
normal	20.2 (103/511)	21.6 (79/366)	11.4 (30/264)
moderate	78.3 (400/511)	75.5 (276/366)	78.8 (208/264)
severe	1.6 (8/511)	3.0 (11/366)	9.8 (26/264)
**Comorbidity**			
diabetes	34.8 (23/66)	15 (22.7/66)	42.4 (28/66) *
hypertension	22.7 (63/278)	25.2 (70/278)	52.1 (145/278) *
ischemic heart disease	34.2 (55/161)	27.4 (44/161)	38.4 (62/161)
generalized pain	31.4 (69/220)	28.6 (63/220)	40.0 (88/220) *
depression	28.2 (11/39)	30.8 (12/39)	41.0 (16/39) *

## Data Availability

The dataset supporting the conclusions of this article is available on request to the corresponding author.
